# The Prevalence and Utilization of Prehospital IV Access in Critically Ill Patients in the Emergency Department

**DOI:** 10.7759/cureus.44111

**Published:** 2023-08-25

**Authors:** Anas Khan, Raed M Alojayri, Naif Alhoseini, Faisel AlZahrani, Saad S Dammas, Mohammed Alothmani, Mohammad Almanjomi

**Affiliations:** 1 Emergency Medicine, King Saud University, Riyadh, SAU

**Keywords:** trauma, ctas, utilization, iv access, ems, prehospital

## Abstract

Background: Despite the pivotality of emergency medical services (EMS) in prehospital care for patient stabilization, prehospital intravenous (IV) access, a standard practice, remains an ambiguity in Saudi Arabia in terms of its prevalence of placement, justification, and utilization.

Objectives: In this study, we aim to estimate the prevalence and utilization rate of prehospital IV access placement in patients transported to King Khalid University Hospital (KKUH) Emergency Medicine Department in Riyadh by EMS and determine the relationship between the prevalence and utilization rate of prehospital IV access in Canadian Triage and Acuity Scale (CTAS) levels 1 and 2 in trauma and non-trauma patients.

Methods: This observational cross-sectional study was conducted over six months. A total of 181 cases of CTAS levels 1 and 2 adult patients were included. Data were collected by trained nurses using convenient sampling through an author-developed questionnaire.

Results: The prevalence of prehospital IV line placement was 28.7%, with a utilization rate of 50%, and was notably higher among CTAS level 1 cases (69.2%). Additionally, trauma cases had a higher prevalence of prehospital IV access (53.5%) compared to medical cases (odds ratio (OR): 4.73, 95% confidence interval (CI): 4.73, 4.73, p<0.05). Among patients with prehospital IV lines, the majority (92.3%) were patent and functional. Upon arrival, 73.1% of patients had their prehospital IV line replaced, with hospital protocol being the most common reason for the replacement (73.7%).

Conclusion: A minority of the patients had prehospital vascular access, and of those, half remained unused. Trauma cases and CTAS level 1 patients had a higher prevalence and utilization of prehospital IV access. Furthermore, trauma cases were more associated with prehospital IV access establishment and utilization.

## Introduction

Emergency medical services (EMS) is a specialized area of medicine that focuses on providing prehospital care to patients in emergency situations. This includes stabilizing patients, administering appropriate treatment, and transporting them to hospitals using ambulances or helicopters. The aim of EMS certification is to establish a uniform standard for healthcare professionals practicing this field, enhance patient safety, and improve the quality of emergency medical care given in prehospital settings. Additionally, this certification helps to integrate prehospital patient management into the overall continuum of care [1].

Over the past few decades, EMS has evolved significantly, with advances in technology and medicine leading to better training, equipment, and protocols. Nonetheless, there are still challenges that must be addressed [1,2]. In the Kingdom of Saudi Arabia (KSA), EMS has witnessed tremendous development since it was established in 1934, notably when the Saudi Board of Emergency Medicine (EM) was formed in 2005 [3,4]. As for the medical care provided by EMS, there are two distinct approaches: "stay and play" and "scoop and run." The stay-and-play approach, commonly implemented in Franco-German systems, focuses on more comprehensive care in the field at the cost of delayed transportation to the hospital. On the contrary, the scoop-and-run approach, which is widely adopted in United Kingdom and United States systems, prioritizes rapid delivery by providing primary and minimum prehospital care, as reducing the time from injury to definitive care can result in a favorable effect on the outcome. Although different rationales back the stay-and-play and scoop-and-run approaches for medical care by EMS, evidence from analyzing the success rates of both approaches shows that scoop-and-run is more effective especially for trauma patients, as literature has not shown an advantage for on-site Advanced Life Support (ALS) provided for them. Additionally, prompt hospital arrival leads to a higher survival rate [5,6].

Prehospital care refers to the medical care provided to patients prior to their arrival at a healthcare facility. Depending on the magnitude of the injury, patients need varying levels of hospital and prehospital care. Thus, a reliable sorting system is needed to assess the urgency and determine the appropriate level of care for each patient. With that being said, there are three values that should be taken into consideration when developing a triage system: the worthiness of human life, healthcare resources, and equitable distribution. A reliable triage system would uphold these values [2,7].

The Canadian Triage and Acuity Scale (CTAS), extensively adopted in KSA, represents a structured five-level sorting system designed to evaluate the level of urgency in medical cases and subsequently assign the most suitable level of care. In this system, the urgency levels are categorized as follows: level 1 for cases requiring immediate resuscitation, level 2 for emergent situations, level 3 for cases of high urgency, level 4 for less pressing situations, and level 5 for non-urgent cases. As emergency departments experience continuing pressures in the aspects of volume and capacity, certain populations are at risk for prolonged wait times. These include elderly sick patients, cognitively impaired, and individuals with disabling conditions. Moreover, it is crucial to minimize the use of any ineffective and unnecessary procedures. Prehospital care ranges from oxygen administration and mild bleeding control to more advanced care such as intravenous (IV) drug or fluid administration, endotracheal intubation, and other invasive interventions [7-9]. The evidence on the prehospital placement of IV access is conflicting, with some studies finding that it is associated with a reduction in mortality, while others finding the exact opposite result [10-12]. Despite that, IV access placement is associated with a higher delivery delay compared to no access, which in turn is associated with increased mortality [11-14]. Many IV accesses were not utilized, which may result in wasting valuable minutes that could be the difference between life and death [15-17].

The requirement and utilization of prehospital IV access in critically ill patients have generated considerable controversy, particularly when considering non-trauma cases. Conversely, numerous guidelines, including the American College of Surgeons' Advanced Trauma Life Support (ATLS) and the Clinical Practice Guidelines of Emergency Medical Services in Saudi Arabia, advocate for the implementation of prehospital IV access in trauma patients. This disparity stems from the intricate nature of patients' presentations, among other contributing factors, which have fueled the ongoing debate. However, the utilization of prehospital IV access has been subject to restrictions due to a growing body of evidence highlighting potential adverse consequences associated with its use [14,18,19]. It is noteworthy to mention that the Clinical Practice Guidelines of Emergency Medical Services in Saudi Arabia specifically recommend the placement of prehospital IV access in traumatic patients, as well as in individuals with special medical conditions such as hypoglycemia and obstetric emergencies. These guidelines serve as a framework to guide healthcare providers in making informed decisions regarding the administration of prehospital care. However, the translation of these guidelines into actual clinical practice may vary, and the extent to which they are adhered to remains uncertain [19].

In KSA, prehospital IV access prevalence and utilization remain unknown compared to areas of a similar population. This may have resulted in patients receiving suboptimal care during the prehospital phase. Understanding the barriers to properly implementing EMS prehospital care is vital to ensure that the best care is administered in emergencies. With the lack of studies regarding EMS prehospital care in KSA, it is crucial to conduct more studies to evaluate and improve EMS prehospital care [3,4]. In this study, we aim to objectively study the prevalence and utilization of prehospital IV access and compare the findings between trauma and non-trauma patients.

## Materials and methods

This observational cross-sectional analytical study was conducted at King Khalid University Hospital (KKUH) Emergency Medicine Department from December 2022 to the end of May 2023. Over six months, the study included 181 patients transported to KKUH Emergency Medicine Department by the EMS. The inclusion criteria included adults (16 years old and more) of CTAS level 1 or 2 transported to KKUH Emergency Medicine Department by EMS from the field. CTAS levels were assessed upon patients' arrival by the KKUH Emergency Medicine triaging team. The data was collected by convenient sampling; trained emergency medicine resuscitation unit nurses administered an author-developed survey. A pilot study was conducted to ensure the reliability of the questionnaire.

Gathered data were analyzed using Statistical Package for the Social Sciences (SPSS) version 24.0 (IBM SPSS Statistics, Armonk, NY, USA), and a p-value of <0.05 and 95% confidence interval (CI) was used to report the statistical significance and precision of the results. Descriptive statistics (mean, standard deviation, frequencies, and percentages) were utilized to describe quantitative and categorical variables, and bivariate statistical analysis was carried out using appropriate tests such as Chi-square test. The study variables include ages, different CTAS levels, the establishment of prehospital IV access, and different patient case types, which were classified into two categories of trauma: medical cases mixed with trauma and isolated medical non-trauma cases. In contrast, the outcome variables include the prevalence, utilization, and replacement of prehospital access.

Ethical considerations have been followed, The Institutional Review Board (IRB) of the College of Medicine, King Saud University, approved the study (project number: E-23-7508). All data collected remained anonymous, and general measures and hospital protocols were adhered to.

## Results

The demographics of the study population were examined in terms of age, sex, acuity level, and case type. The results showed that the age range of 25-44 years had the highest representation, with 56 (31%) patients, followed by 16-24 years (28 patients, 15.5%) and 50-60 years (27 patients, 14.9%). The mean age was 50.80, with a standard deviation of 22.5. The sex distribution demonstrated that male patients were slightly more prevalent than female patients, accounting for 59.7% (n=108) and 40.3% (n=73) of the population, respectively. Regarding acuity level, most patients were CTAS level 2 (146 patients, 80.7%), while the remaining were CTAS level 1 (35 patients, 19.3%). Finally, medical cases accounted for 76.2% (n=138) of our population, while the remaining cases were either trauma or mixed cases with medical and trauma conditions (Table 1).

**Table 1:** Prevalence and utilization rate of prehospital IV access in trauma and non-trauma patients. IV: intravenous, CTAS: Canadian Triage and Acuity Scale

		IV line (number (%))		Utilization (number (%))		Case type (number (%))	
		Yes (52 (28.7%))	No (129 (71.3%))	Yes (26 (50%))	No (26 (50%))	Medical (138 (76.2%))	Trauma/mixed (43 (23.8%))
Age (number (%))							
	16-24 (28 (15.5%))	11 (39.30%)	17 (60.70%)	6 (54.5%)	5 (45.5%)	12 (42.9%)	16 (57.1%)
	25-34 (26 (14.4%))	11 (42.30%)	15 (57.70%)	3 (27.3%)	8 (72.7%)	16 (61.5%)	10 (38.5%)
	35-44 (30 (16.6%))	14 (46.70%)	16 (53.30%)	7 (50%)	7 (50%)	21 (70%)	9 (30%)
	45-54 (15 (8.3%))	4 (26.70%)	11 (73.30%)	4 (100%)	0 (0%)	11 (73.3%)	4 (26.7%)
	55-64 (27 (14.9%))	6 (22.20%)	21 (77.80%)	3 (50%)	3 (50%)	24 (88.9%)	3 (11.1%)
	65-70 (8 (4.4%))	3 (37.50%)	5 (62.50%)	2 (66.7%)	1 (33.3%)	8 (100%)	0 (0%)
	71-80 (23 (12.7%))	2 (8.70%)	21 (91.30%)	1 (50%)	1 (50%)	22 (95.7%)	1 (4.3%)
	80+ (24 (13.3%))	1 (4.20%)	23 (95.80%)	0 (0%)	1 (100%)	24 (100%)	0 (0%)
Gender (number (%))							
	Male (108 (59.7%))	38 (35.20%)	70 (64.80%)	20 (52.6%)	18 (47.4%)	77 (71.3%)	31 (28.7%)
	Female (73 (40.3%))	14 (19.20%)	59 (80.80%)	6 (42.9%)	8 (57.1%)	61 (83.6%)	12 (16.4%)
CTAS (number (%))							
	Level 1 (35 (19.3%))	13 (37.1%)	22 (62.9%)	9 69.2%	4 30.8%	27 (77.1%)	8 (22.9%)
	Level 2 (146 (80.7%))	39 (26.7%)	107 (73.3%)	17 (43.6%)	22 (56.4%)	111 (76%)	35 (24%)

The prevalence of prehospital IV access was observed in 28.7% (n=52) of patients, while the remaining 71.3% (n=129) did not have prehospital IV access. The prevalence of prehospital IV access was found to be higher in CTAS level 1 patients (37.1%, 13/35) compared to CTAS level 2 patients (26.7%, 39/146) and in trauma cases (53.5%, 23/43) compared to medical cases (21%, 29/138). Most prehospital IV lines were patent and functional (92.3%, 24/26). Upon arrival at the hospital, 73.1% (38/52) of patients had their prehospital IV line replaced, while 26.9% (14/52) did not. The majority of replacements were due to hospital protocol (73.7%, 28/38), followed by the need for a larger bore IV catheter (7.9%, 3/38), infiltration (7.9%, 3/38), dislodgement (5.3%, 2/38), and non-patency (5.3%, 2/38). These results provide insights into the prevalence and management of prehospital IV access in emergency medical services (Table 2).

**Table 2:** Prevalence of prehospital IV access in terms of different case types, patency, replacement, and justification for the replacement. IV: intravenous

		IV line (number (%))	
		Yes (52 (28.7%))	No (129 (71.3%))
Case type (number (%))			
	Medical (138 (76.2%))	29 (21%)	109 (79%)
	Trauma/mixed (43 (23.8%))	23 (53.5%)	20 (46.5%)
Patent (number (%))			
	Yes	24 (92.3%)	NA
	No	2 (8%)	NA
Replaced (number (%))			
	Yes	38 (73%)	NA
	No	14 (27%)	NA
Justification for the replacement (number (%))			
	Hospital protocol	28 (73.7%)	NA
	Infiltrated	3 (7.9%)	NA
	Dislodged	2 (5.3%)	NA
	Need for a larger bore IV	3 (7.9%)	NA
	Not patent	2 (5.3%)	NA
Prehospital medication administered (number (%))			
	Nothing was given	8 (15.7%)	NA
	IV fluid	39 (76.5%)	NA
	Aspirin	2 (3.9%)	NA
	Midazolam	1 (1.9%)	NA
	Morphine	1 (1.9%)	NA

The utilization rate of prehospital IV access was examined among patients who had a prehospital IV line, and it was found that 50% (n=26) of these patients had their IV line utilized. The utilization of prehospital IV access was higher in CTAS level 1 patients (69.2%, 9/13) compared to CTAS level 2 patients (43.6%, 17/39). Most patients with prehospital IV access received IV fluid (76.5%, 39/51), while smaller proportions received midazolam (11.8%, 6/51) or morphine (11.8%, 6/51). It is important to note that the medications and fluids mentioned here only include those given in the prehospital setting and do not include anything administered after the patient arrives at the hospital (Table 1).

The prevalence and utilization rate of prehospital IV access in trauma and non-trauma patients were examined in the study. The results showed that 21% (n=29) of patients had prehospital IV access in medical cases, while 79% (n=109) did not. In contrast, 53.5% (n=23) of patients had prehospital IV access in trauma cases, compared to 46.5% (n=20) who did not. The study showed a significantly higher prevalence of prehospital IV access in trauma cases compared to medical cases (odds ratio (OR): 4.73, 95% CI: 4.73, 4.73, p<0.05). On the contrary, a significantly lower prevalence of prehospital IV access in medical cases was observed when compared to trauma cases (OR: 0.51, 95% CI: 0.51, 0.51, p<0.05). Moreover, among patients with prehospital IV access, the rate of IV line utilization was 44.8% (n=13) in medical cases and 56.5% (n=13) in trauma cases. However, the difference was not statistically significant (OR: 0.50, 95% CI: 0.24, 1.06, p>0.05). In contrast, the odds ratio for prehospital IV access utilization was 2.42 (95% CI: 1.20, 4.87, p>0.05) in trauma cases, indicating a non-significant trend toward higher utilization rates of prehospital IV access compared to medical cases (Table 3).

**Table 3:** Prevalence and utilization rate of prehospital IV access in trauma and non-trauma patients. IV: intravenous, CI: confidence interval

IV line prevalence (number (%))		Odds ratio	95% CI	Chi-square value (df)	p-value
	Medical (29 (21%))	0.51	0.51, 0.51	16.89 (1)	0.0001
	Trauma/mixed (23 (53.49%))	4.73	4.73, 4.73		
Utilization (number (%))		Odds ratio	95% CI	Chi-square value (df)	p-value
	Medical (13 (44.82%))	0.50	0.5, 0.5	0.70 (1)	0.421
	Trauma/mixed (13 (56.5%))	2.42	2.42, 2.42		

## Discussion

The aim of this cross-sectional analytical study was to investigate the prevalence and utilization of prehospital IV access placement in different CTAS levels and different case types. The results revealed that a minority of patients arrived at the hospital with prehospital IV access, while the majority did not. The prevalence of prehospital IV access was higher in CTAS level 1 patients compared to CTAS level 2 patients and in trauma cases compared to medical cases. Our findings are consistent with a Swiss study that reported peripheral vascular access placement in 26% of cases transported [15]. Another study conducted in the United States in 2014 focused on the prehospital management of patients with severe sepsis and found that 23% received prehospital IV catheter and fluid administration, while 7% had a catheter alone, and 70% had neither a catheter placed nor fluid administered [20].

The authors hypothesize that trauma cases are, on average, more likely to receive IV access as ATLS recommends keeping the vein open by inserting an IV or interosseous line in trauma patients because they have a higher tendency to lose blood, which needs to be compensated promptly [18]. Our results showed that more than half of the trauma cases received an IV line in the prehospital setting versus only a small minority in pure medical cases, comparing that with a study from California in which the records of 34,585 patients who were transported through an ambulance in a county with a population of around 700,000 were collected, and the prevalence was as low as 7.7%. The study also demonstrated that males are more likely than females to have an IV line placed; however, no difference was noted in terms of its utilization. The study by Kuzma et al. noted that age played a role, as children were half as likely to receive an IV line as adults; this could be explained by the fact that children are less likely to suffer more injuries and that they only constituted 3.3% of the study's population. However, the utilization was equal in both age groups [16]. Another prospective study from Pierce County enrolled 290 subjects and found that 23% of trauma cases arrived at the hospital with an IV line [21]. On the other hand, a 2013 study examining the impact of IV access on prehospital times and time to the first packed red blood cell (PRBC) transfusion in trauma patients concluded that as much as 81% received prehospital IV access.

As for the prevalence, utilization rates were also variable throughout the literature. A study in the United States found that 83% of the IV lines were unutilized [16]. Comparing that with another study conducted in France on the feasibility of IV line placement, the figure was only 29% [22]. In this study, we found that half of the prehospital IV lines were unutilized, which is closely comparable to a study in Switzerland in which 45.9% were unutilized [15]. Furthermore, we found that most CTAS level 1 patients had their IV access utilized. This could suggest that the more severe the presentation, the more likely the patient would get IV access. As mentioned before, trauma cases are more likely to have IV lines placed and are more likely to be utilized. Our results suggest that the utilization rate is higher in trauma cases, closely comparable to what the Kuzma et al. study reported (40%) of medical non-trauma cases. Pace et al. reported only a 10% utilization rate. On the contrary, their report showed much higher prevalence (74%) and utilization (37%) in medical cases [21]. The higher percentages in medical cases of the latter study go against the authors' hypothesis; however, it is somewhat accepted as it could be attributed to the different guidelines and the change in trend over the years. Moreover, the nature of the medical cases reported (i.e., hypotension) is more likely to receive and use IV lines than cases such as stroke [16].

Importantly, the utilization of the study findings and their implementation into clinical practice might be a challenge, as some literature highlights defects in the application of research outcomes into practice and policy [23]. This gap in evidence and clinical practice was discussed in several studies [24] that used the BARRIERS scale, which was implemented on emergency medical technicians (EMTs) to assess for this gap. The BARRIERS scale is a scale used to assess the perceptions of barriers to the use of research evidence in clinical practice. Samarkandi et al. reported the top three barriers to be as follows, ranked from top to bottom: implications for practice are not made clear, the relevant literature is not compiled in one place, and the EMT feels that the benefits of changing practice will be minimal [24]. With that being said, Samarkandi et al. showed that these barriers exist between different health professions, including paramedics, emphasizing the importance of assessing them based on specific situations or contexts [24].

The conflicting results in the literature could be attributed to different approaches to prehospital IV access, such as the "scoop-and-run" and "stay-and-play" strategies. In addition, the decision to initiate a prehospital IV access depends on multiple variables, including primary impression, the nature of the injury, the patient's initial vital signs, and the CTAS level or any other trauma triage system. Presently, the Clinical Practice Guidelines of Emergency Medical Services in Saudi Arabia advocate for the implementation of prehospital IV access specifically in trauma patients, with a key emphasis on minimizing any potential delay in transportation that may arise from the cannulation process. Additionally, these guidelines recommend the utilization of prehospital IV access in a limited subset of medical conditions.

Limitation

This study is subject to some limitations that should be considered. Firstly, the data collection and analysis did not comprehensively account for potential confounding variables that may contribute to the observed prevalence and utilization of prehospital IV access. Factors such as underlying comorbidities were not adequately addressed, which could potentially influence the findings and limit the ability to draw definitive conclusions.

Secondly, it is noteworthy to mention the relatively small scale of this study, encompassing a total of 181 patients over a span of six months. This limited sample size of EMS transports could potentially impact the robustness and generalizability of the results, an aspect that should be taken into account during interpretation.

Lastly, the investigation focused solely on the prevalence and utilization patterns of prehospital IV access among different CTAS levels and case types, without simultaneous evaluation of relevant patient outcomes, including morbidity and mortality. Incorporating outcome measures would provide a more comprehensive understanding of the overall implications associated with prehospital IV access and enhance the interpretation of the findings within a broader clinical context.

## Conclusions

A minority of our patients had prehospital vascular access. Of those, half remained unused. Trauma cases and CTAS level 1 patients had a higher prevalence and utilization of prehospital vascular access. Furthermore, trauma and mixed cases were more associated with prehospital IV access establishment and utilization.
